# Update and New Insights on Future Cancer Drug Candidates From Plant-Based Alkaloids

**DOI:** 10.3389/fphar.2021.719694

**Published:** 2021-12-16

**Authors:** Mounir Tilaoui, Hassan Ait Mouse, Abdelmajid Zyad

**Affiliations:** Experimental Oncology and Natural Substances Team, Cellular and Molecular Immuno-pharmacology, Faculty of Sciences and Technology, Sultan Moulay Slimane University, Beni-Mellal, Morocco

**Keywords:** cancer, plant-based alkaloids, preclinical research, molecular mechanism, clinical trials

## Abstract

Cancer is a complex multifactorial disease that results from alterations in many physiological and biochemical functions. Over the last few decades, it has become clear that cancer cells can acquire multidrug resistance to conventional anticancer drugs, resulting in tumor relapse. Thus, there is a continuous need to discover new and effective anticancer drugs. Natural products from plants have served as a primary source of cancer drugs and continue to provide new plant-derived anticancer drugs. The present review describes plant-based alkaloids, which have been reported as active or potentially active in cancer treatment within the past 4 years (2017–2020), both in preclinical research and/or in clinical trials. In addition, recent insights into the possible molecular mechanism of action of alkaloid prodrugs naturally present in plants are also highlighted.

## 1 Introduction

Cancer remains a leading cause of death worldwide. The survival rate, morbidity, recurrence, and poor prognosis of the disease have been the major challenges for decades in traditional nonsurgical cancer therapy regimens, including chemotherapy and radiotherapy (Zhang et al., 2020d). Nevertheless, the survival rate of patients diagnosed with cancer, particularly those in advanced stages, remains at very low levels due to drug resistance, high toxicity, and other long-term side effects of these treatments (Zhang et al., 2020d; Zhang et al., 2020a). This has encouraged scientists to search for more effective strategies and discover new anticancer drugs.

Natural products, especially those from plants, have been an extraordinarily important source for new drug discovery over the past decades (Berdigaliyev and Aljofan, 2020). At present, plant-based drugs are becoming a major source of cancer treatment (Rahman et al., 2020). Many of them have demonstrated significant anti-tumor effects, such as flavonoids (Khan et al., 2020), phenylpropanoids (Hematpoor et al., 2018), lactones (Tilaoui et al., 2014), taxanes, epipodophyllotoxins, and alkaloids (Zishan et al., 2017). In this context, there have been many studies on the anticancer activity of alkaloids due to their higher specificity, stronger effectiveness, and lower toxicity (Lu et al., 2012). This review mainly focuses on the recent cumulative preclinical research and ongoing clinical trials on alkaloids originating from plants that have been reported as effective or potentially effective in cancer treatment, with emphasis on their molecular mechanisms of action. This may provide a deep understanding of the signaling pathways to identify and develop a safe novel lead compound that selectively targets cancer cells and improves therapeutic applications for cancer treatment and prevention. The literature search was performed through the PubMed database to identify recent published original research between 2017 and 2020.

## 2 Alkaloids in the Plant Kingdom

**FIGURE 1:**
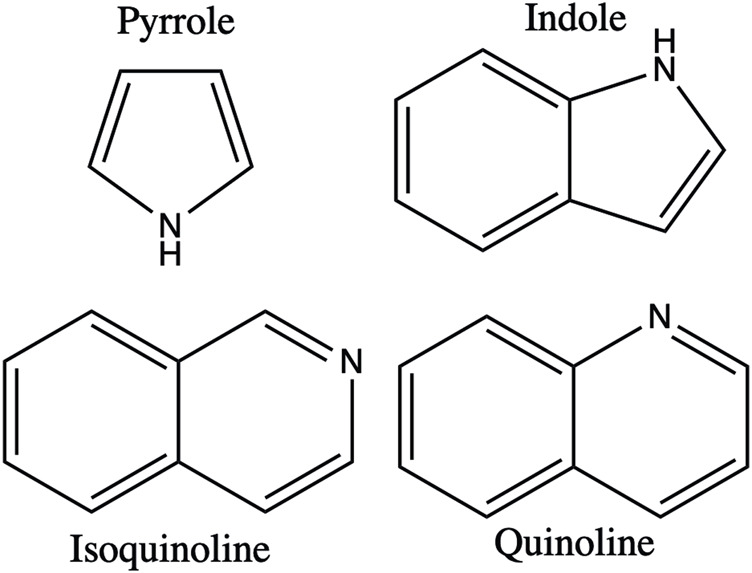
Basic structure of alkaloids.

Alkaloids are a widespread class of secondary metabolites that have animal, microbial, and plant origins. They contain a nitrogen atom and a ring structure. The position of the nitrogen atom in the carbon ring varies with different alkaloids (Figure 1). Alkaloids are predominantly derived from simple amino acid precursors, such as tyrosine, phenylalanine, tryptophan, ornithine, arginine, or lysine. Today, more than 20,000 compounds are known to encompass a large variety of chemical structures with different functional groups (Table 1), and approximately 12,000 different alkaloids have been identified in the plant kingdom, mainly in higher plants, and are distributed into various classes based on their chemical structures, biosynthetic pathways, and biological activities (Dey et al., 2020). Furthermore, many alkaloids exhibit a wide range of biological activities, including antimicrobial, antimalarial, antifungal, and anticancer effects (Debnath et al., 2018). In fact, many plant-derived alkaloids have been successfully approved as chemotherapeutic drugs by the US Food and Drug Administration (FDA), such as vinblastine, which interacts with tubulin to interfere with the cell cycle (Li et al., 2007; Choudhari et al., 2020), and camptothecin, a known potent topoisomerase I inhibitor (Huang et al., 2007).

**TABLE 1:** Functional groups of plant-based alkaloids.

Alkaloid	Structure	Functional group
Nitidine chloride	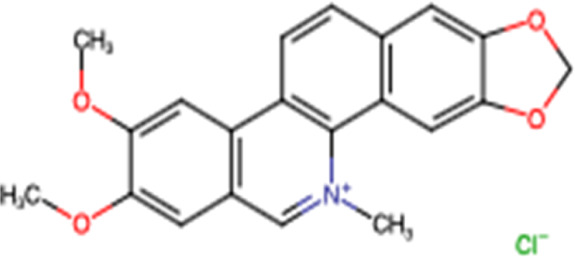	Arene
		Benzene ring
		Acetal
		Ether
Sophoridine	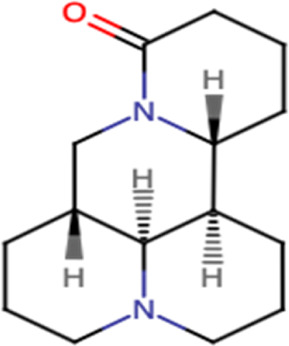	Amine, tertiary
		Carboxamide
		Carboxamide, tertiary
		Lactam
		Carbonyl
Palmatine	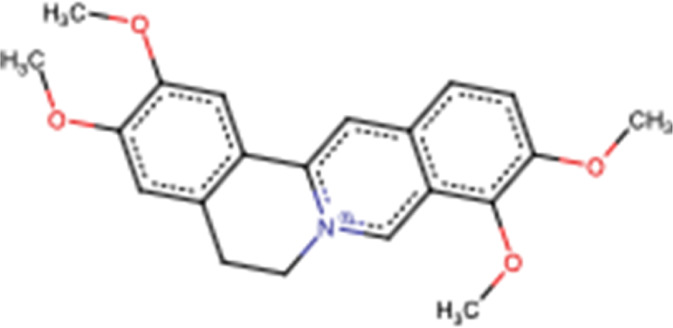	Arene
		Benzene ring
		Ether
Aleutianamine	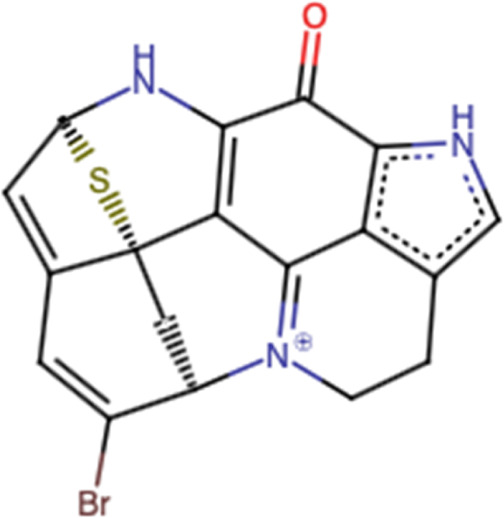	Arene
		Azaarene
		Heteroarene
		Alkene
		Amine
		Amine, secondary
		Enamine
		Α,β-unsaturated carbonyl
		Carbonyl
		Ketone
		Aldehyde
		Alkenyl bromide
		Alkenyl halide
		Leaving group
		Sulfide
Nuciferine	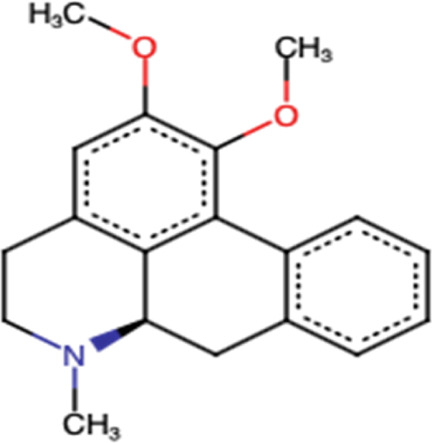	Arene
		Benzene ring
		Amine
		Amine, tertiary
		Ether
Theobromine	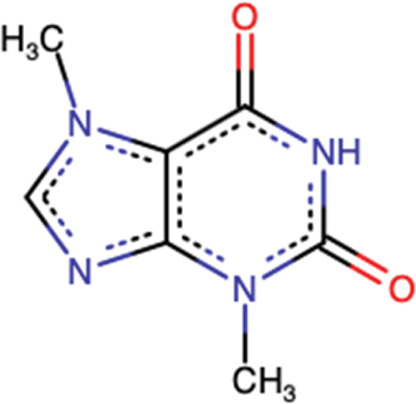	Arene
		Azaarene
		Heteroarene
		Amine
		Amine, secondary
		Amine, tertiary
Berberine	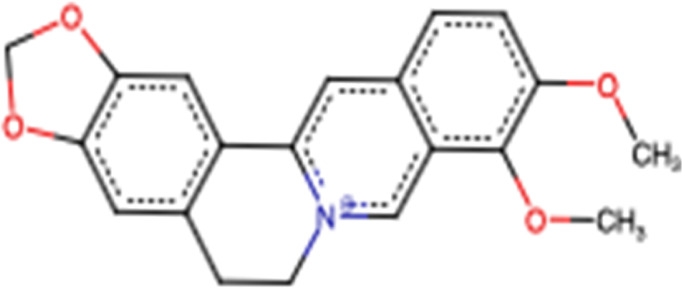	Arene
		Benzene ring
		Acetal
		Ether
Homoharringtonine	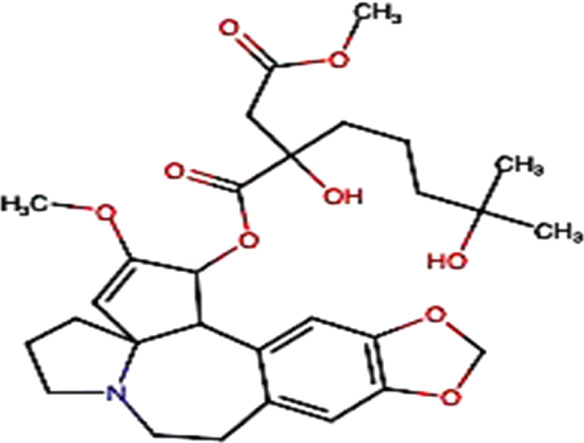	Arene
		Benzene ring
		Alkene
		Amine
		Amine, tertiary
		Acetal
		Alkanol
		Carbonyl
		Enol ether
		Ester (carboxylate ester)
		Ether

## 3 Anticancer Plant-Based Alkaloids: Preclinical Research

### *3.1 Annona muricata* L Alkaloids

The alkaloids isolated from the root of *Annona muricata* L are traditionally used in Indonesia to treat various diseases. An unreported benzylisoquinoline alkaloid, (+)-xylopine 5, was isolated from this plant-based source and demonstrated significant cytotoxic effects compared to the four known alkaloids (coclaurine, reticuline, argentinine, and atherospeminine) against the human suspension cancer cell line (HL-60 leukemia cells) and two human cancer cell lines (A549 lung cancer cells and HepG2 liver cancer cells) with IC_50_ values ranging from 20 to 80 µM (Nugraha et al., 2021). According to the US National Cancer Institute, a natural compound has potent anticancer activity if it has an IC_50_ value around or below 10 μM or 4 μg/ml ( Boik 2001; Kuete and Efferth 2015; Kuete et al., 2018 ).

### 3.2 Nitidine Chloride

**FIGURE 2:**
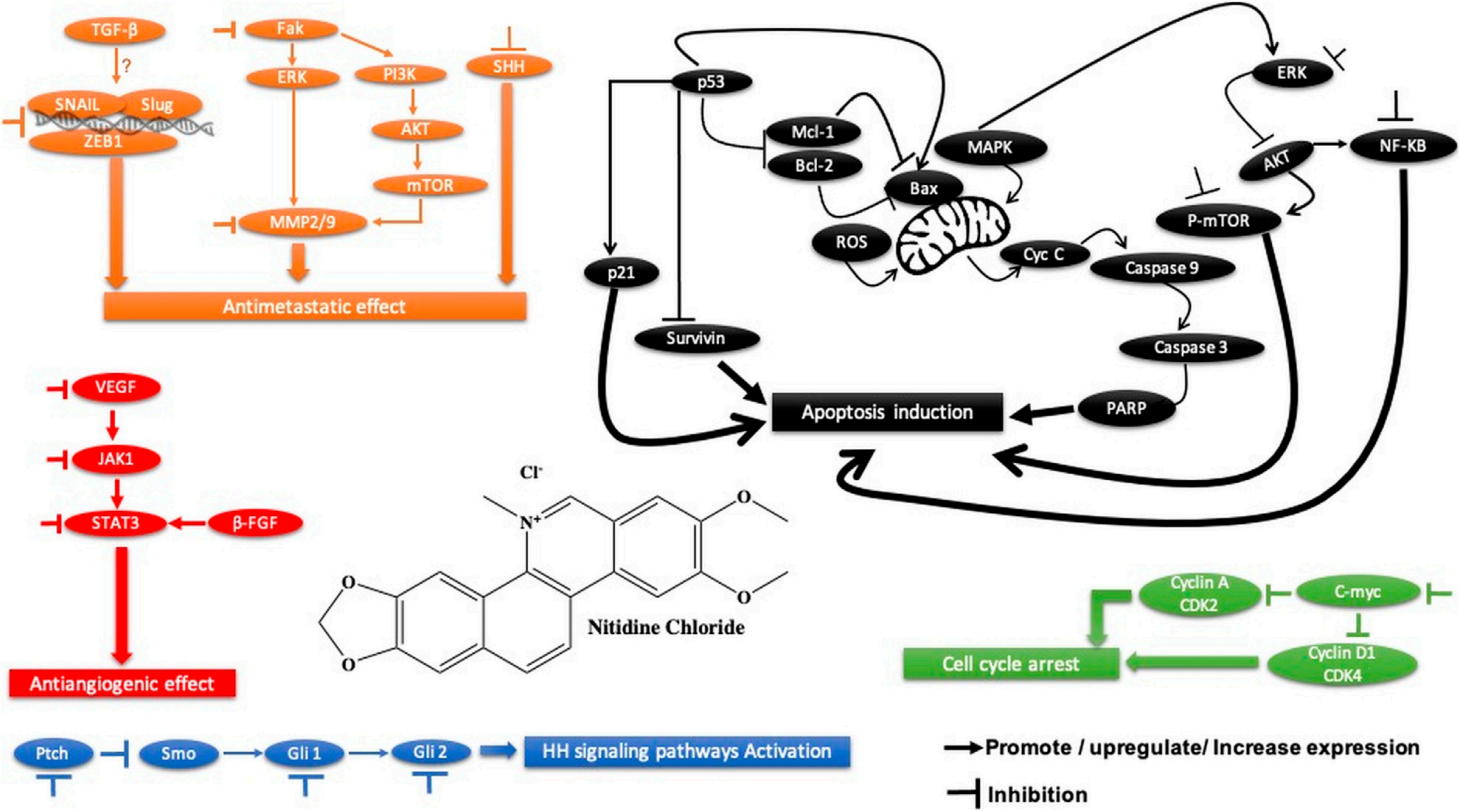
Main mechanism of nitidine chloride against cancer.

Nitidine chloride, a major active ingredient isolated from the roots of the traditional Chinese medicinal herb *Zanthoxylum nitidum* (Roxb) DC, has been demonstrated to have anticancer effects on human malignant tumors, including HCC (Table 2). Xiong et al., 2019 demonstrated that nitidine chloride inhibits proliferation by G2/M phase arrest and induces apoptosis (Figure 2) of HCC cells *in vitro* with an IC_50_ of 1.332 μM/L in SMMC7721 cells and 5.006 μM/L in Huh7 cells. In addition, it has been shown that nitidine chloride exerts antimetastatic effects by suppressing cell migration and invasion (Figure 2). In *in vivo*, nitidine chloride has been shown to decrease the tumor volume with a total of 297 circRNAs differentially expressed between the nitidine chloride-treated and nitidine chloride-untreated groups. Thus, circRNAs may play an important role in the progression of hepatocellular carcinoma (Xiong et al., 2019).

**TABLE 2:** Summary of recent preclinical and clinical trials of plant-based alkaloids on cancer treatment.

Alkaloid type	Preclinical research		Clinical trial	Reference
	*In vitro* studies	*In vivo* studies		
Nitidine chloride	Liver cancer: Huh7 (IC_50_ = 5.006 μM); SMMC7721 (IC_50_ = 1.332 μM)	Liver cancer: Tumor volume decreased in BALB/c nude mice injected with SMMC7721	Not applicable	Xiong et al. (2019)
Sophoridine	Lung Cancer: H460 ((IC_50_ = 73.49 μg/ml/24 h); (IC50 = 53.52 μg/ml/48 h)); Lewis ((IC_50_ = 64.95 μg/ml/24 h); (IC50 = 40.49 μg/ml/48 h))	Lung cancer: 15 or 25 mg/kg suppressed the tumor growth and upregulated the expression of CD86/F4/80 in tumor tissues in the Lewis-bearing mice model	Not applicable	Peng et al. (2020)
	Gastric cancer: SGC7901 (IC_50_ = 3.52 μM/12 h); AGS (IC_50_ = 3.91 μM/12 h); GES-1 (IC_50_ = 51.40 μM/12 h)			Zhao et al. (2021)
Palmatine	Colon cancer: HT-29 (IC_50_ = 68.3 µM); SW-480 (IC_50_ = 72.6 µM)	Colon cancer: Gavage once a day with 33.75, 67.5, and 135 mg/kg inhibits the growth of HCT-116 xenograft tumors; −10 to 20 mg/kg/day decreased tumor numbers in the small intestine and colon in ApcMin^/^+ mice	Not applicable	Johnson-Ajinwo et al. (2019)
	Ovarian cancer: IC_50_ values ranging from 5.5 to 7.9 µM			Tuzimski et al. (2021)
	Melanoma: A375 (IC_50_ > 200 μg/ml); G361 (IC_50_ = 119.98 μg/ml); SK-MEL-3 (IC_50_ = 88.04 μg/ml)			Hambright et al. (2015)
	Prostate cancer: DU145 (IC_50_ = 10 μg/ml)			Liu et al. (2020a)
	Gastric cancer: MKN-45 (IC_50_ = 332.43 µM)			Ma et al. (2016)
				Yu et al. (2020)
Aleutianamine	Pancreatic cancer: PANC-1 (IC_50_ = 25 nM)	Not applicable	Not applicable	Zou et al. (2019)
	Colon cancer: HCT-116 (IC_50_ = 1 μM)			
Nuciferine	Lung cancer: A549/T (IC_50_ = 105.1 μM); A549 (IC_50_ = 129.4 μM)	Lung cancer: Moderate tumor growth inhibition in the A549/T xenograft model in BALB/c-nu/nu mice treated with 7.5 mg/kg/ip/3 days for 27 days	Not applicable	Li et al. (2019)
	Glioblastoma: U87MG (IC_50_ = 72.3 μM); U251 (IC_50_ = 59.9 μM)	Glioblastoma: Inhibition of tumor volume growth in glioblastoma xenograft BALB/c nude mouse models		Liu et al. (2020b)
	Breast cancer: 60 μM reduced cell viability to 40% in MDA-MB-231 and 20% in MCF-7 cells			Kang et al. (2017)
	Liver cancer: 100 μM reduced cell viability to 60% in HepG2 and Huh7; and 40% in HCClM3			
	Cervical carcinoma: 100 μM reduced cell viability of HeLa to 45%			
	Colon carcinoma: HCT-8/T (IC_50_ = 104.79 μM); HCT-8 (IC_50_ = 164.16 μM)			
	Lung cancer: A549/T (IC_50_ = 105.1 μM); A549 (IC_50_ = 129.4 μM) (Z. Li et al., 2019)			
Methylxanthines	Breast cancer: MDA-MB-231 (IC_50_ > 100 μM, Theophyline)	Breast cancer: Not applicable	Breast cancer: Study phase not applicable^a^; (NCT03482401)	Hashemi-Niasari et al. (2018)
	Gastric cancer: MGC-803 (IC_50_ = 4 mM, Caffeine); MGC-803 (IC_50_ = 8 mM, Theophylline)	Gastric cancer: BALB/c nude mice injected intraperitoneally with caffeine (4 mM) or theophylline (8 mM) results in reducing tumor size		Liu et al. (2019a)
	Oral epidermoid carcinoma: KB (IC_50_ = 2.5 mM/48 h)			Ávila-Gálvez et al. (2019)
	Lung cancer: H1355 (IC_50_ = 2.5 mM/48)			Caraglia et al. (2002)
	Melanoma: GLL-1 (IC_50_ = 2.5 mM/48)			Beklen et al. (2021)
	Colon carcinoma: HCT-116 (IC_50_ = 500 μM/72 h)			
Berberine	Colon cancer: SW620 (IC_50_ = 54.41 μM/48 h); LoVo (IC_50_ = 78.66 μM/48 h)	Colon cancer: Dose of 10 mg/kg/qd/ip/for 2 weeks reduces tumor volume in Balb/c mice	Colorectal adenoma: Phase II, Phase III; NCT02226185	Zhao et al. (2017)
	Breast cancer: BT549 (IC_50_ = 16.575–1.219 mg/ml); MDA-MB-231 (IC_50_ = 18.525–6.139 mg/ml)	Breast cancer: Oral dose (100 mg/kg)/3 days inhibited tumor growth and increased caspase-9 levels in MDA-MB-231 in the BALB/c mouse-xenograft model; −0.1% in the drinking water/day during 6.6 w promoted the antitumoral activity in female Balb/c; −50 mg/kg/po decreased the tumor size in rats		Kim et al. (2018)
	Glioblastoma: U87 (IC_50_ = 42 μM); U251 (IC_50_ = 32 μM)	Glioblastoma: 50 mg/kg berberine reduced tumor weight and improved the survival rate of mice in the ectopic tumor xenograft mouse model and inhibits angiogenesis in glioblastoma xenografts		Karnam et al. (2017)
	Ovarian cancer: OVCAR3 (IC_50_ = 99 μM, 24 h)	Nasopharyngeal carcinoma: 10 mg/kg/ip/qd for 3 weeks decreases the tumor volume in female NOD/SCID mice		Liu et al. (2019b)
		Gastric cancer: Antitumoral activity at 50 mg/po/qd during 4 weeks in Balb/c mice		Jin et al. (2018)
		Neuroepithelial tumor: Treatment with 10 mg/kg/qd/ ip during 1 week showed antitumor effect in Balb/c mice		Ruan et al. (2017)
		Endometrial carcinoma: Antitumoral activity at 50 mg/kg/po during 4 weeks in Balb/c mice		Wang et al. (2017a)
				Wang et al. (2016)
				Sun et al. (2018b)
				Wang and Zhang (2018)
				Liu et al. (2015)
Homoharringtonine	Breast cancer: MDA-MB-157 (IC_50_ = 15.7 ng/ml, 24 h); MDA-MB-468 (IC_50_ = 19.9 ng/ml, 24 h); CAL-51 (IC_50_ = 23.1 μg/ml, 24 h); MDA-MB-231 (IC_50_ = 80.5 ng/ml, 24 h); MDA-MD-231 (IC_50_ = 0.31 μg/ml); HCC 1937 (IC_50_ = 0.32 μg/ml); T47D (IC_50_ = 1.27 μg/ml); MCF7 (IC_50_ = 0.45 μg/ml)	Breast cancer: 1 mg/kg given subcutaneously, twice daily, over 7 days suppresses growth of MDA-MB-231 and MDA-MB-468 in Swiss nu/nu female mice; −50 μg/kg/day injected intraperitoneally for 10 days inhibited breast cancer cell growth in female and male BLAB/c nude mice	Hematologic malignancies solid tumors: Phase I; NCT01844869	Wang et al. (2021)
	Liver cancer: HepG2 (IC_50_ = 0.025 μM); Bel-7402 (IC_50_ = 0.251 μM); Hep3B (IC_50_ = 0.291 μM); Bel-7404 (IC_50_ = 0.694 μM); SMMC-7721 (IC_50_ = 1.220 μM)	Liver cancer: 0.1 mg/kg, 0.2 mg/kg, and 0.4 mg/kg administered orally decreased tumor growth and the inhibitory rate of 20, 50, and 55% in mal nude mice, respectively	Leukemia: Phase III; NCT00004933	Yakhni et al. (2019)
	Acute myeloid leukemia: MV4-11 (IC_50_ = 0.15 nM/24 h; 5.32 nm/48 h); MOLM13 (IC_50_ = 6.06 nM/24 h; 1.54 nM/48 h); CCL-AML2 (IC_50_ = 65 nM/24 h; 4.67 nM/48 h); CCL-AML3 (IC_50_ = 23.94 nM/24 h; 4.85 nM/48 h)	Acute myeloid leukemia: 1.0 mg/kg/day by oral administration for 2 weeks suppressed AML progression and cell growth in the xenograft female NOD/SCID mice model	Acute myelogenous leukemia: Phase II; NCT01873495	Zhu et al. (2020)
			Hematologic tumors: Phase I; NCT00675350	Shi et al. (2021)
			Leukemia: NCT02159872; Phase II	
			Chronic myeloid leukemia: Phase I/II; NCT02078960	

IC_50_: Half maximal inhibitory concentration.

a Study phase not applicable describes trials without Food and Drug Administration (FDA)–defined phases.

NCT: Clinical trial umber.

#### 3.2.1 Nitidine Chloride Safety

Importantly, nitidine chloride has a well-established safety; it shows no nephrotic and no hepatic toxicity at a dose of 10 mg/kg in mouse (Kim et al., 2017, 3). Also, no acute toxicity was observed within CD female mice treated intraperitoneally with doses ranged from 0.1 to 20 mg/kg (LD_50_ = 20 mg/kg) (Bouquet et al., 2012).

### 3.3 Sophoridine

**FIGURE 3:**
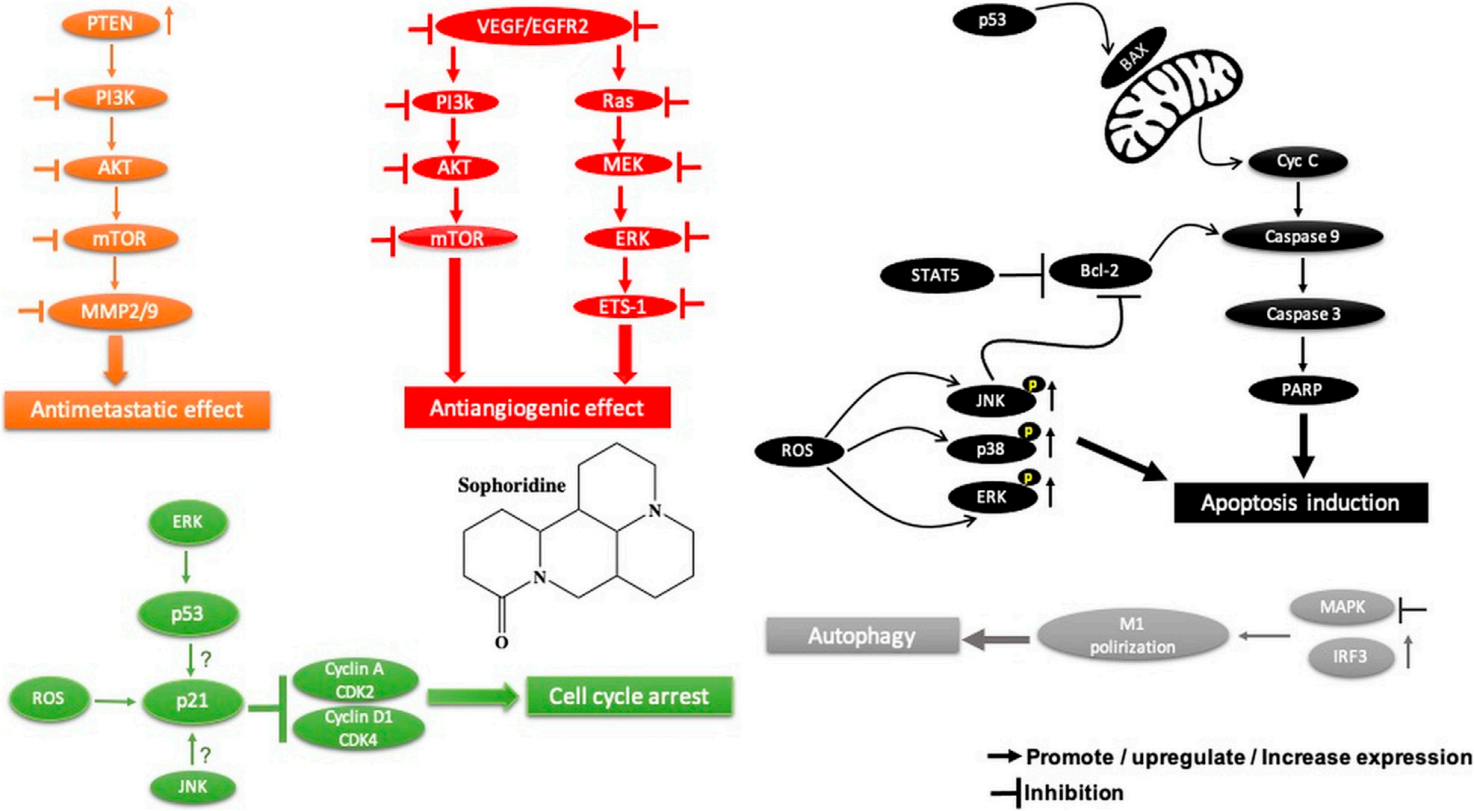
Main mechanism of sophoridine against cancer.

Sophoridine is a quinolizidine-based alkaloid extracted from the leaves and stems of *Euchresta japonica Benth* and *Sophora alopecuroides* L. and roots of *Sophora alopecuroides* Ait. plants. It is known to exhibit various pharmacological effects, including anti-inflammatory, anti-anaphylactic, antiarrhythmic, and antiviral activities (ur Rashid et al., 2020). Sophoridine has been shown to exert potent cytotoxic activity against gastric cancer cells (Table 2) by suppressing the polarization of M2 tumor–associated macrophages, increasing the polarization of M1-tumor–associated macrophages through the TLR4/IRF3 pathway, and inhibiting tumor-associated macrophage infiltration by downregulating the expression of CCR2 in the gastric cancer microenvironment, subsequently enhancing the cytotoxic function of CD8^+^ T cells and alleviating CD8^+^ T cell function exhaustion (Zhuang et al., 2020) (Figure 3). Another study showed that sophoridine significantly inhibits survival, invasion, and migration of gastric cancer cells by enhancing estrogen-related receptor gamma expression, which leads to the degradation of β-catenin. Furthermore, it induces G2/M cell cycle arrest by inhibiting double-stranded DNA break repair and enhances the efficacy of cisplatin in gastric cancer cells (Peng et al., 2020). Therefore, sophoridine may be a promising candidate for human gastric cancer treatment. In terms of potential toxicity, there is, however, no safety assessment of sophoridine has been reported yet to the best of our knowledge.

### 3.4 Palmatine

**FIGURE 4:**
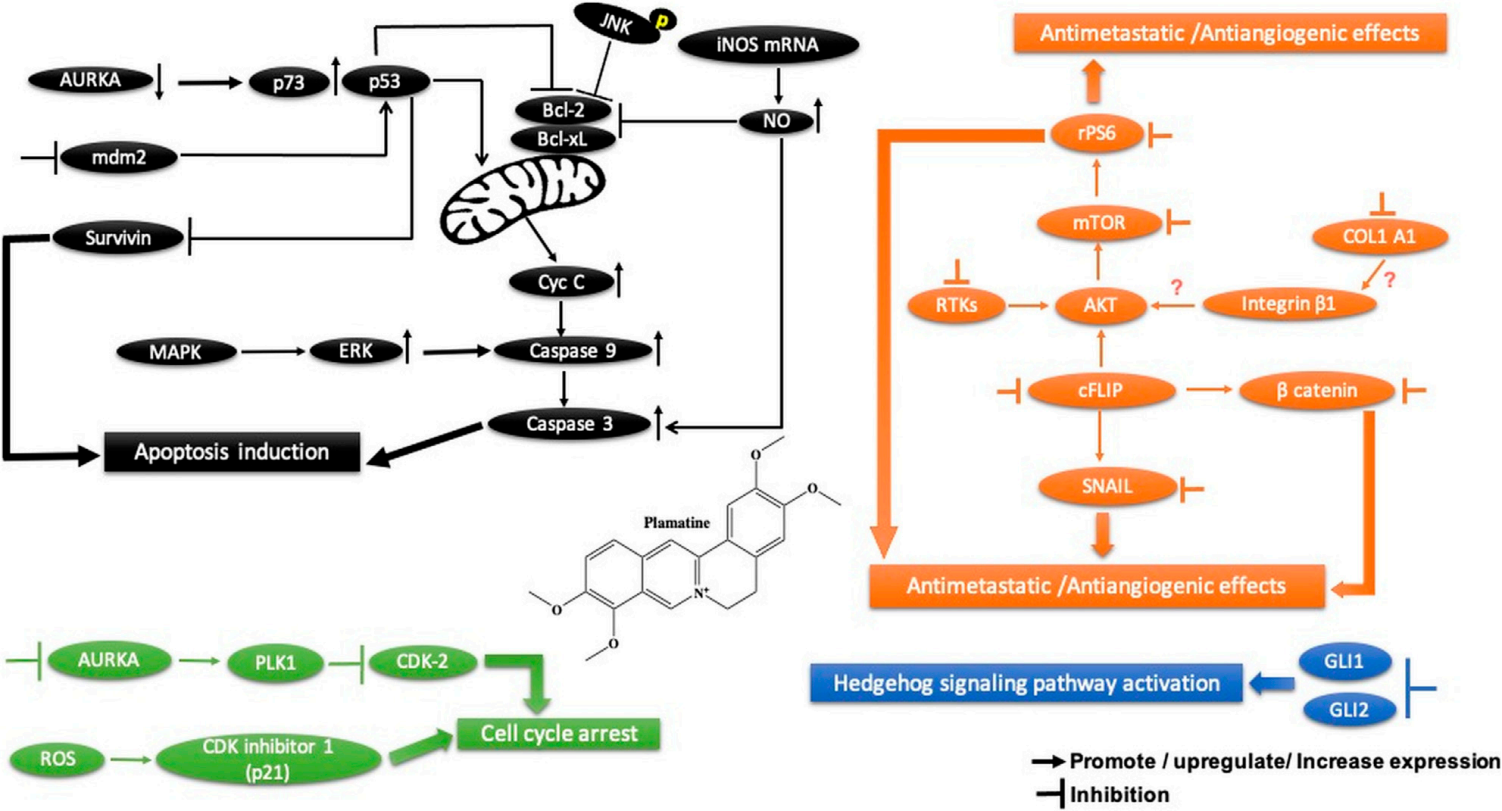
Main mechanism of palmatine against cancer.

Palmatine, another alkaloid from *Rutidea parviflora*, has been shown to exhibit strong cytotoxic activity against ovarian cancer cell lines with IC_50_ values ranging from 5.5 to 7.9 µM (Table 2). Importantly, palmatine is less cytotoxic toward immortalized human ovarian epithelial cells. Furthermore, it induces apoptosis by increasing caspase 3-7 activity and poly(ADP-ribose) polymerase cleavage in OVCAR-4 cancer cells (Johnson-Ajinwo, Richardson, and Li 2019) (Figure 4). Additionally, palmatine has been shown to inhibit proliferation and migration while inducing apoptosis by inhibiting the survivin protein in pancreatic cancer cells, either alone or in combination with the conventional drug gemcitabine. Chakravarthy et al. linked these effects to the suppression of glutamine-mediated changes in the GLI signaling pathway in pancreatic cancer cells, which thereby induces apoptosis with simultaneous inhibition of survivin and collagen type 1 alpha 1 (Chakravarthy et al., 2018, 1).

#### 3.4.1 Palmatine Safety

On the evidence available, palmatine appears to be a safe drug in animal models. In mice, the acute toxicity showed that the LD_50_ is 1,533.68 mg/kg. In the subchronic toxicity, no adverse effects, no mortality, and no morbidity were revealed in rats treated with 156 mg/kg for 90 days (Yi et al., 2013).

### 3.5 Aleutianamine

**FIGURE 5:**
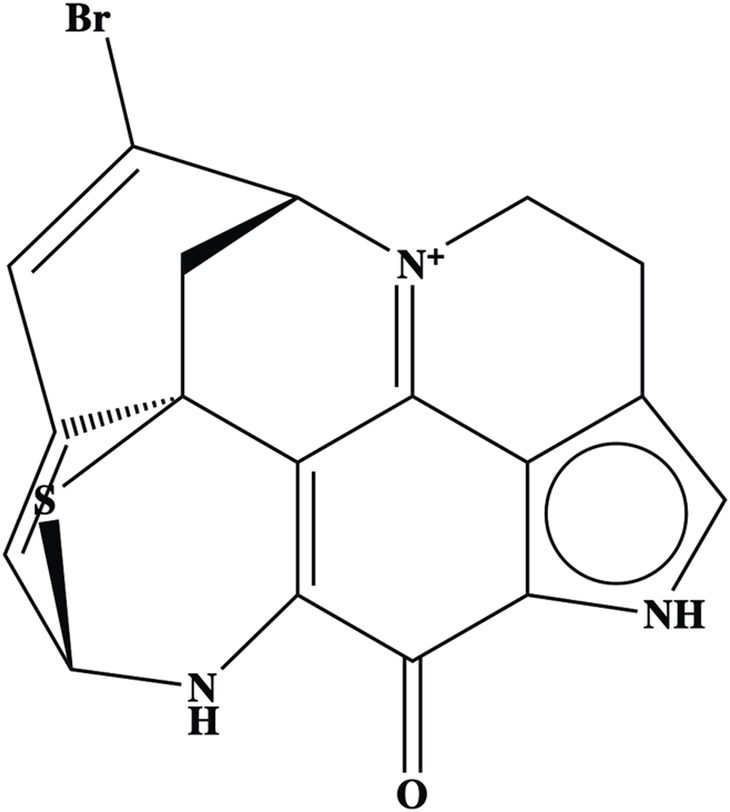
Structure of aleutianamine.

Aleutianamine (Figure 5), a new class of pyrroloiminoquinone alkaloids from Alaska’s deep ocean, was found to exhibit potent and selective cytotoxicity toward solid tumor cell lines, including pancreatic cancer (PANC-1), with an IC_50_ of 25 nM and colon cancer (HCT-116) with an IC_50_ of 1 μM (Zou et al., 2019). In terms of safety, to our knowledge, no scientific reports about the potential toxicity of aleutianmine have been investigated.

### 3.6 Nuciferine

**FIGURE 6:**
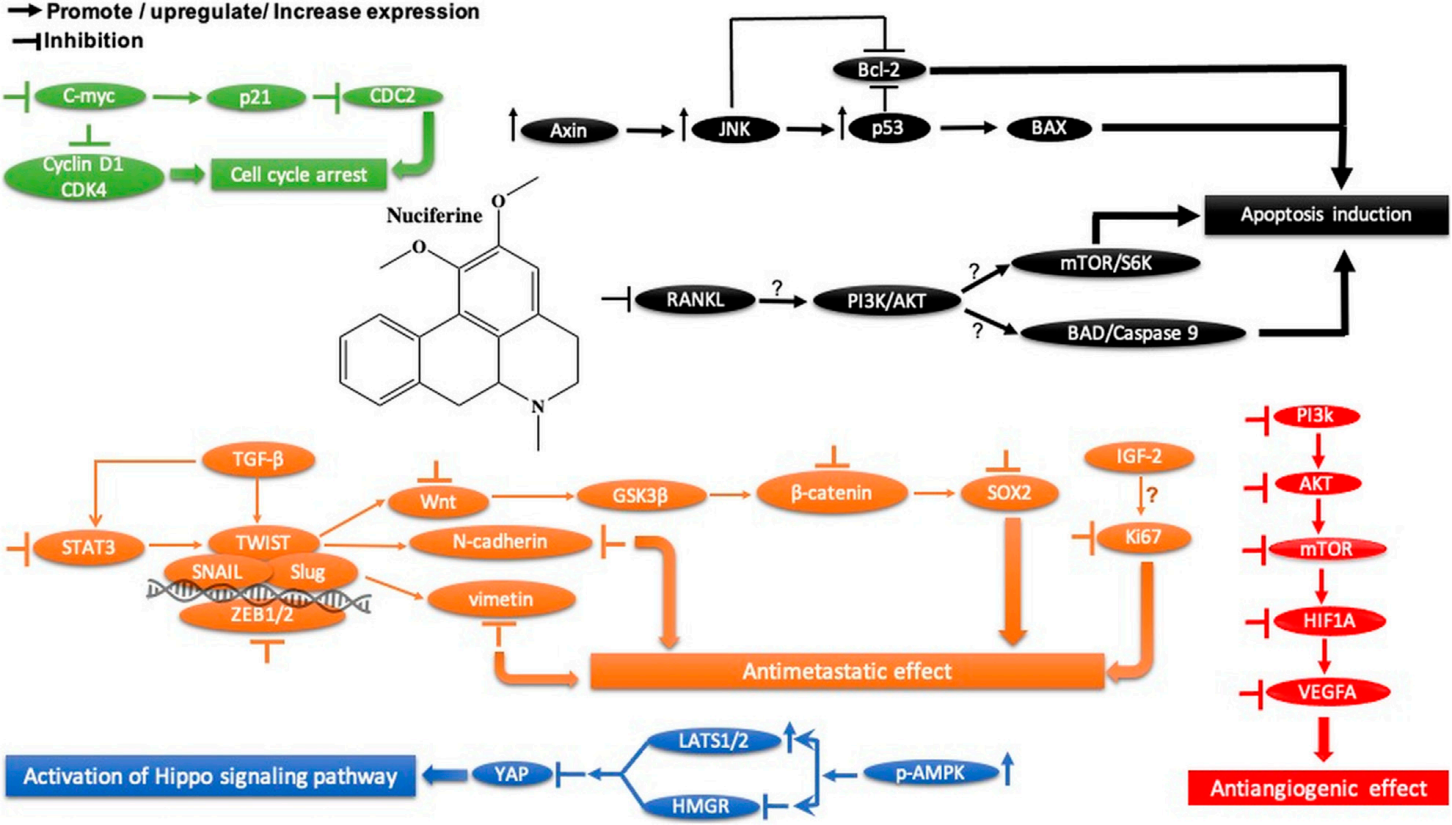
Main mechanism of nuciferine against cancer.

Nuciferine, extracted from the leaves of *Nelumbo nucifera Gaertn*, has been shown to inhibit cell migration and angiogenesis by inducing apoptosis and G2 cell cycle arrest in glioblastoma cells (U87MG and U251). These effects are triggered by the decrease in Slug expression through the SOX2-AKT and STAT3 signaling pathways (Figure 6). Interestingly, it showed less cytotoxicity in normal human cells (HUVECs, LO2, and HK2). In *in vivo*, nuciferine exhibited significant tumor control in glioblastoma xenograft BALB/c nude mouse models. Therefore, nuciferine could be a potential drug for the treatment of glioblastoma. In addition, nuciferine has been found to have potent cytotoxic activity (Table 2) against breast cancer (MDA-MB-231 and MCF7), hepatocellular carcinoma (HepG2, Huh7, and HCCLM3), and cervical carcinoma (HeLa) cell lines in a dose-dependent manner (Z. Li et al., 2019).

#### 3.6.1 Nuciferine Safety

Nuciferine has been found to be a promising anticancer drug. However, its toxicity should not be ignored. Previous experimental studies have shown that the median lethal doses (LD_50_) of nuciferine were 240 mg/kg, po and 280 mg/kg, po in mice and rat, respectively (Bhattacharya et al., 1978; Soleymankhani et al., 2015). Also, another report indicated that nuciferine induced significant hypotension after intravenous injection of 4–10 mg/kg and 10–20 mg/kg in cats and dogs, respectively (Neuwinger, 1996).

### 3.7 *Corydalis yanhusuo W.T. Wang* Alkaloids

**FIGURE 7:**
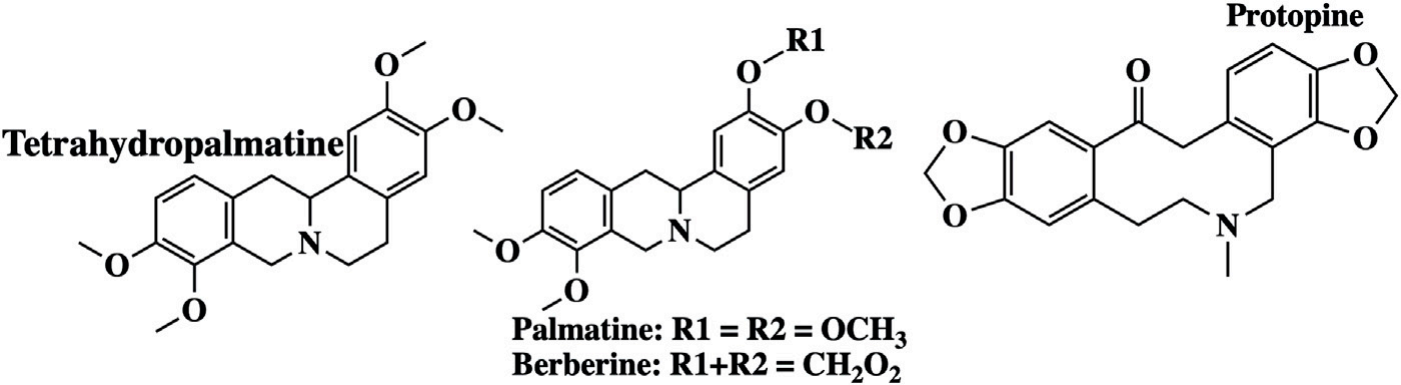
Examples of molecular structures of alkaloids obtained from *Corydalis yanhusuo*.

*Corydalis yanhusuo* W.T. Wang (*Papaveraceae*) is a well-known Chinese herbal plant widely used for various pharmacological properties, especially to improve blood circulation, alleviate pain caused by blood stasis, and reinforce vital energy (J. Zhang et al., 2020b). These biological activities are mainly attributed to their alkaloid constituents (Figure 7) (Tian et al., 2020). In a recent study, alkaloid fractions of *Corydalis yanhusuo* W.T. Wang demonstrated potent inhibition of a variety of VEGF-induced angiogenic processes such as proliferation, sprouting, migration of endothelial cells, HUVECs, and decreased blood vessel formation in both Matrigel plug of mice and chick chorioallantoic membrane models. Wan et al. (2019) concluded that the mechanism underlying these effects is related to the suppression of the VEGF-induced signaling pathway through VEGFR2 phosphorylation diminution, which leads to downstream phospho-ERK1/2, phospho-AKT, and phospho-STAT3 levels in HUVECs (Wan et al., 2019). In another study, Kośmider et al. (2017) evaluated the cytotoxic effect of the tetracyclic alkaloid–free aqueous extract of *Uncaria tomentosa* leaf against cancer HepG2 cells. The results from this study show the differential effects of these alkaloids on cancer and normal NHDF cells. It exhibits significant cytotoxic activity in HepG2 cells by activating caspase 3 and caspase 9 compared to that in normal cells. Furthermore, in cancer cells, NF-κB expression is reduced, which may prevent the development of multidrug resistance to chemotherapy (Kośmider et al., 2017).

## 4 Anticancer Plant-Based Alkaloids: Clinical Trials

### 4.1 Methylxanthines

#### 4.1.1 Preclinical Studies

Previous research demonstrated that methylxanthines exhibits antitumor activity in animal models and differential cytotoxic effect against cancer cells such as Caco-2, A549, and T24 (Amigo-Benavent et al., 2017). Also, it was reported that methylxanthine derivatives such as theobromine and theophylline effectively suppress MGC-803 gastric cancer cell proliferation and migration, induce apoptosis, and promote autophagy in both *in vivo* and *in vitro* through LC-3, Beclin-1/Bax, and Bcl-2/Bcl-xL regulation, PTEN activation, PI3K-Akt-mTOR signaling pathway suppression, blocking cell migration through the ROCK/FAK pathway and may exert its effects on global *epigenetic* machinery mediated by HAT-histone acetyltransferases, HDAC-2, and HDAC-1, and regulating alternative splicing by targeting SRSF3 (Pérez-Pérez et al., 2019). Furthermore, it has been reported that caffeine, another alkaloid belonging to methylxanthine family (1,3,7-trimethylxanthine), activates p38 MAPK phosphorylation through production of ROS and an increase in the intracellular calcium Ca^2+^ concentration in U937 cancer cells (Cui et al., 2020 ). Moreover, Cheng et al. reported that caffeine decreased the invasion of glioma cancer cells via FAK and ERK signaling pathways (Cheng et al., 2016 ). Also, it exhibits anti-tumor immune response by increasing T lymphocyte infiltration and decreasing PD-1 expression on CD8^+^ T lymphocytes and CD4^+^ and CD25^+^ regulatory T lymphocytes in caffein-treated groups in a carcinogen-induced local fibrosarcomas tumor model (Tej et al., 2019). The anti-tumor effect of caffeine observed in this study is mediated through the release of cytokines TNF-α and IFN-γ. Thus, Tej et al., 2019 suggested that blockade of the adenosine pathway by caffeine can effectively enhance the anti-tumor immune response.

#### 4.1.2 Clinical Studies

**FIGURE 8:**
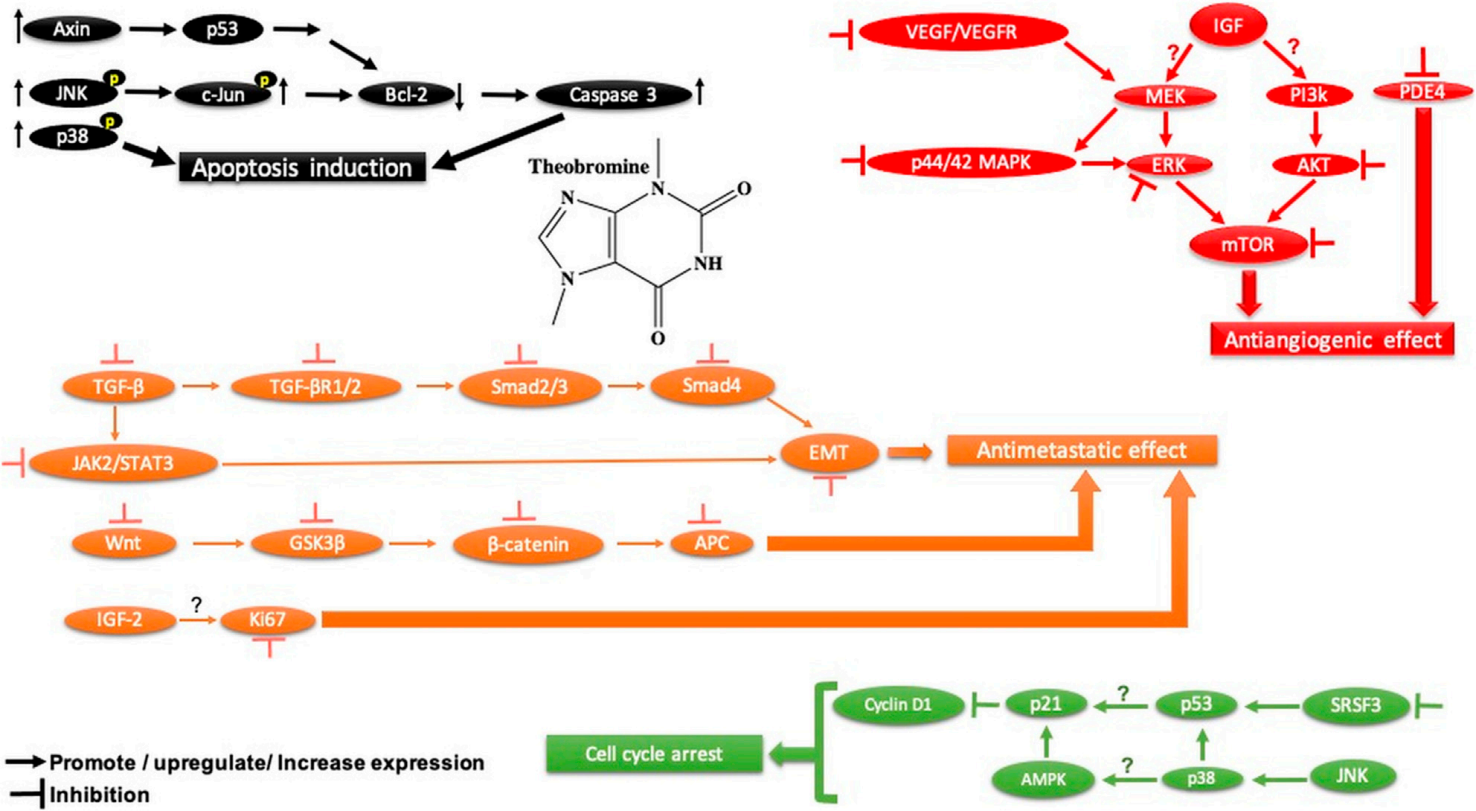
Main mechanism of theobromine against cancer.

Ávila-Gálvez and coworkers showed in a randomized clinical trial (NCT03482401) that the disposition of methylxanthines (theobromine and caffeine) in malignant and normal breast tissues after consumption of dietary methylxanthines including theobromine (19.2 ± 0.6 mg day−1) and caffeine (0.48 ± 0.03 mg day−1) could provide chemoprevention and/or treatment of breast cancer. Numerous mechanistic studies have shown that methylxanthines inhibit polymerase 1 under physiological conditions and reverse multidrug resistance by downregulating the breast cancer resistance protein (ABCG2/BCRP), which may increase the efficacy of anticancer drugs that are the ABCG2/BCRP substrate (Ávila-Gálvez et al., 2019) (Ávila-Gálvez et al., 2019) (Figure 8).

Similarly, it has been reported previously that methylxanthine blocks the nuclear enzyme poly (ADP-ribose)polymerase1 (Geraets et al., 2006), rendering tumor cells more sensitive to chemotherapeutic drugs (Rosendahl et al., 2015) and reverses breast cancer cell resistance to various conventional drugs by downregulating the breast cancer resistance protein (BCRP/ABCG2) (Ding et al., 2012).

#### 4.1.3 Methylxanthine Safety

Methylxanthine toxicity depends on its alkaloid derivative and the animal in question. In the rate, the LD values were 206 mg/kg, 200 mg/kg, and 950 mg/kg for theophylline, caffeine, and theobromine, respectively. In human, the LD values were 1000 mg/kg for theobromine and 192 mg/kg for caffeine. Consequently, human acute toxicity regarding methylxanthines is very low. However, it should be kept in mind that methylxanthine pharmacological concentrations of high doses could cause anxiety, an increase in gastric secretion, and heart rate (Bonetti et al., 2017). Furthermore, it is important to note that methylxanthine derivatives may have a negative impact on pregnancy and general offspring development. In zebrafish embryos, theophylline, caffeine, pentoxifylline, 3-isobutyl-1-methylxanthine, etophylline, aminophylline, and doxofylline were embryotoxic and teratogenic at higher doses. Ref methylxanthines induce structural and functional alterations of the cardiac system in zebrafish embryos. Early studies showed that caffeine intake slightly increases the risk of spontaneous abortion (Weng et al., 2008), increases the risk of intrauterine growth restriction, increases the incidence of congenital limb deficiencies (Chen et al., 2012), and increases the risk of having a baby with cryptorchidism (if the dose exceeds 400 mg/day) (Mongraw-Chaffin et al., 2008), encephalocele, and spina bifida (Schmidt et al., 2009). However, other epidemiological studies reported negative effects on prenatal and pregnancy outcomes associated to maternal ingestion of high and moderate doses of caffeine (Ådén, 2011).

### 4.2 Berberine

#### 4.2.1 Preclinical Studies

**FIGURE 9:**
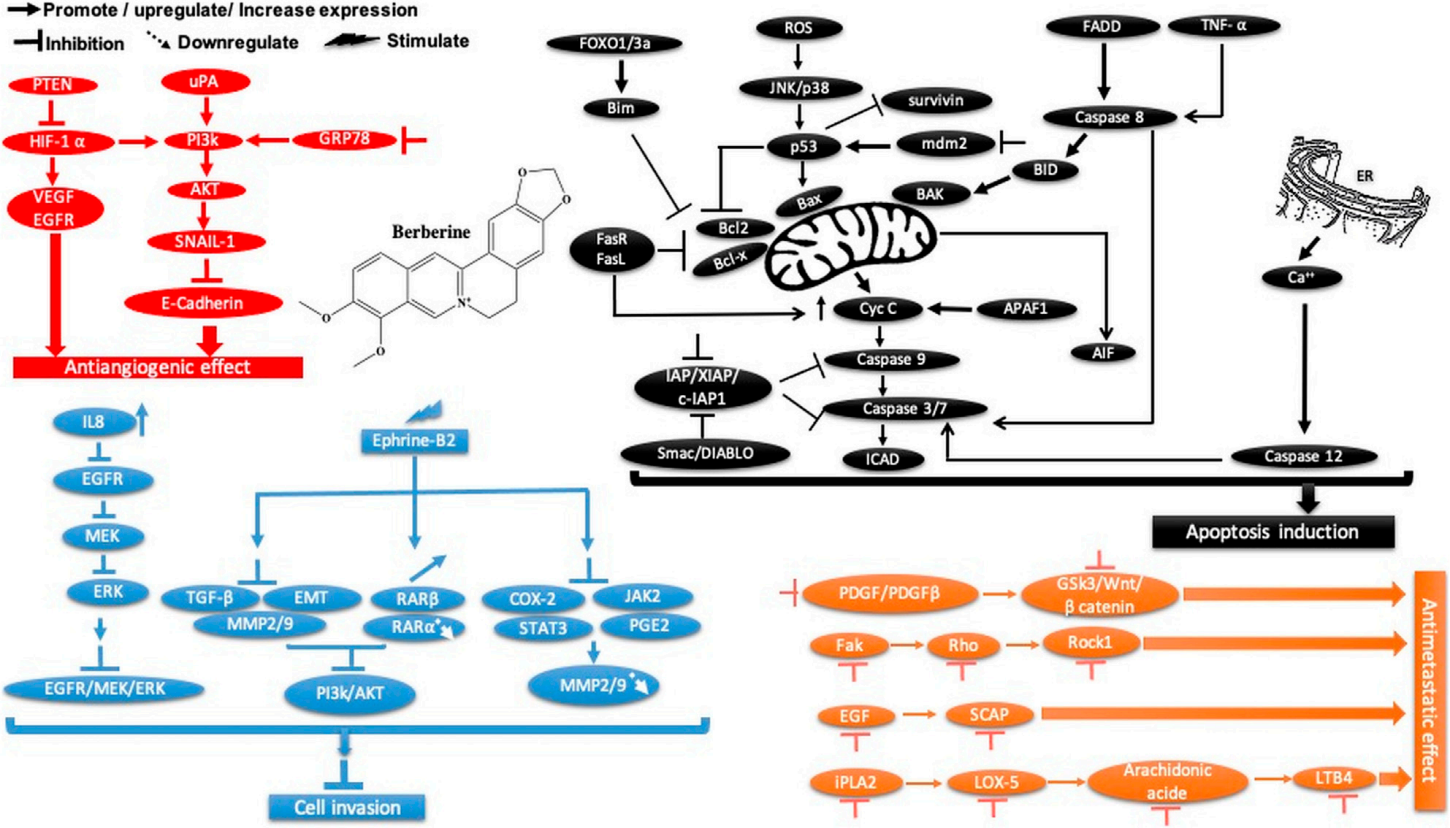
Schematic representation of apoptotic events, cell migration, and angiogenesis inhibition of berberine against cancer.

**FIGURE 10:**
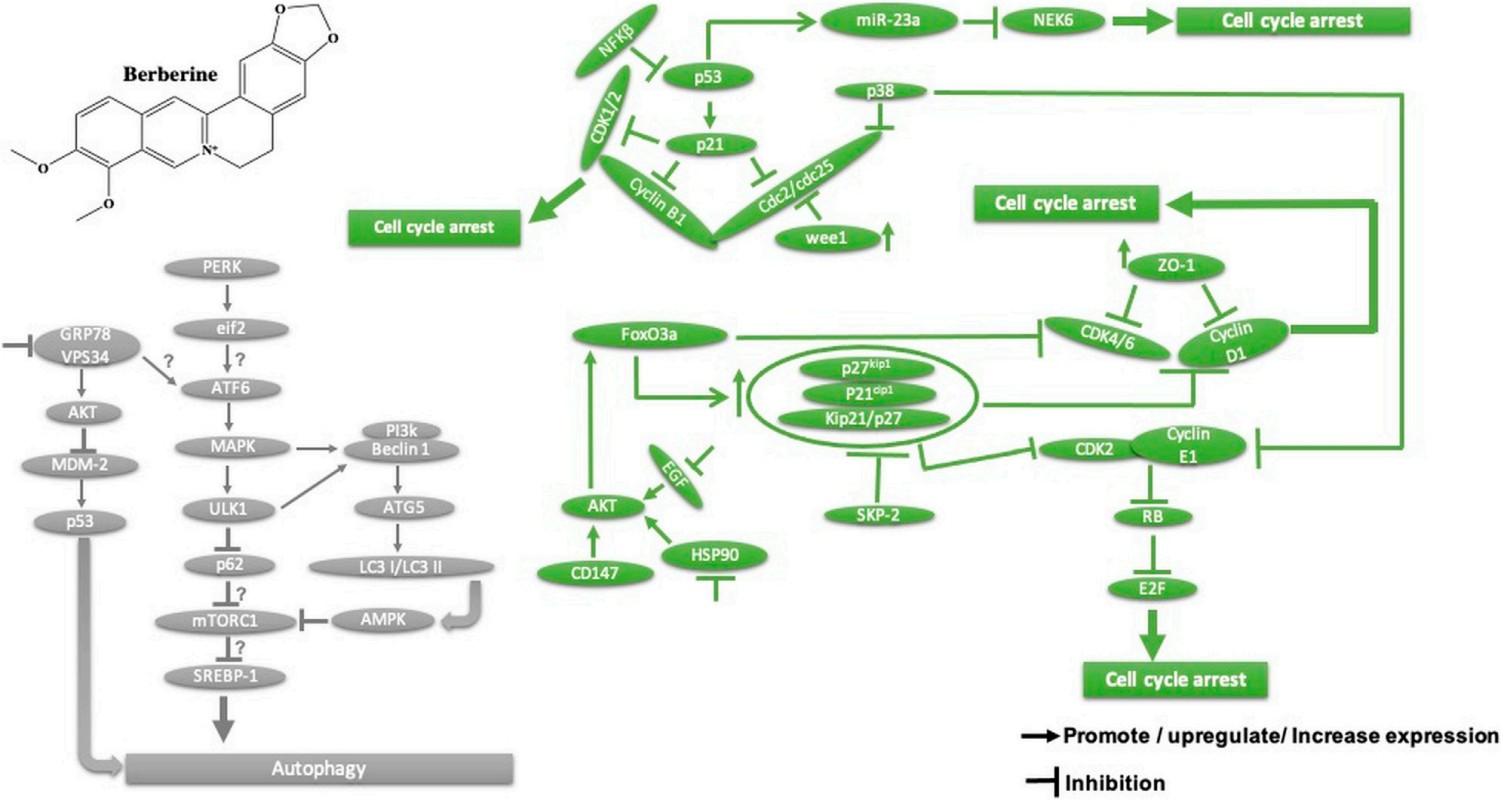
Overview of the main molecular mechanisms of berberine-induced autophagy and cell cycle arrest.

Berberine is another plant-derived alkaloid that has been reported to exhibit *in vitro* and *in vivo* cancer activities. In our lab, berberine from *Berberis vulgaris* was found to exhibit strong cytotoxic activity against the breast cancer cell line MCF-7, with an IC_50_ = 8.75 μg/ml. The cytotoxic effect is attributed to its ability to induce apoptosis without pronounced cytotoxic activity in normal cells. In another study, it exhibited antiproliferative activity, decreased the migration and invasion of gastric carcinoma cells, and suppressed gastric carcinoma tumor growth *in vivo* (Table 2). The anti-gastric carcinoma effect of berberine might be linked to the AMPK/HNF4 α/WNT5A pathway (Hu et al., 2018) (Figure 9). However, Wang and Zhang found that berberine isolated from *Rhizoma coptidis* inhibits endometrial tumor cell motility, invasion, and metastasis *in vitro* and *in vivo* through miR-101-COX2-PGE2 signaling pathways (Figure 9) (Y. Wang and Zhang 2018, 2). In addition, berberine mediated suppression of cell motility by downregulating the transforming growth factor ß-1 in triple-negative breast cancer cells (Kim et al., 2018) and induced apoptosis through activation of caspase 9/cytochrome c, which leads to growth inhibition of TNBC cells in both *in vitro* and *in vivo* assays (Figure 9). In liver cancer, berberine inhibits hepatocellular carcinoma Hep3B and BEL-7404 via suppression of glutamine uptake by inhibiting SLC1A5 and c-Myc both *in vitro* and *in vivo* (P. Zhang et al., 2019). In a recent study, berberine was found to repress human gastric cell proliferation both *in vitro* and *in vivo* by the autophagy process and cell cycle arrest (Figure 10) through blocking cyclins and the Akt and MAPK-mTOR-p70S6K pathways (Q. Zhang et al., 2020c).

#### 4.2.2 Clinical Studies

Moreover, berberine (0.3 g) taken twice daily by oral administration was highly effective against colorectal adenomas, and no colorectal cancers were detected during follow-up. In addition, it was safe, with no serious adverse events. Thus, the authors concluded that berberine could be effectively used to reduce the risk of colorectal adenoma recurrence, polypoid lesions, and as a chemopreventive agent after polypectomy NCT02226185. However, it has been concluded that the mechanism of action of berberine is still unclear (Y.-X. Chen et al., 2020).

#### 4.2.3 Berberine Safety

Berberine can play an important role in prevention and treatment of cancer. However, its toxicity should not be dismissed. Overall, berberine has shown very low toxicity in animal studies (Mohammadzadeh, Mehri, and Hosseinzadeh 2017). Its toxicity depends on the animal species, route of administration, and the dose. In rat, the LD_50_ of berberine is >15,000 mg/kg, and the LD_50_ values in mice are 20.8 g/kg, 57.6 mg/kg, and 9.03 mg/kg through oral, intraperitoneal, and intravenous administration, respectively (Kheir et al., 2010). In human, the dose used to treat metabolic disease ranged from 0.4 to 1.5 g/day (Xu et al., 2021). Some clinical trials have reported mild gastrointestinal adverse reactions, including constipation and diarrhea (Imenshahidi and Hosseinzadeh 2019), and no adverse effects have been seen in the liver (Xu et al., 2021). In high doses, berberine has been associated with dyspnea, flu-like symptoms, cardiac damage, and hypotension (Imanshahidi and Hosseinzadeh, 2008; Imenshahidi and Hosseinzadeh, 2016). Other studies showed that berberine may lead to kernicterus in infants with glucose-6-phosphate dehydrogenase deficiency (Fung and Linn, 2015). Also, berberine can act directly on the fetus development, and it may lead to birth abnormalities. Thus, its use during pregnancy, neonatal, and breastfeeding is cautioned. Conversely, berberine has not been found to cause cytotoxicity, mutagenicity, and genotoxicity with its prescribed clinical doses (Rad et al., 2017). Further preclinical studies on berberine safety on animals and well-designed human clinical trials could better delineate and understand the therapeutic role of berberine in humans.

### 4.3 Homoharringtonine

#### 4.3.1 Preclinical Studies

**FIGURE 11:**
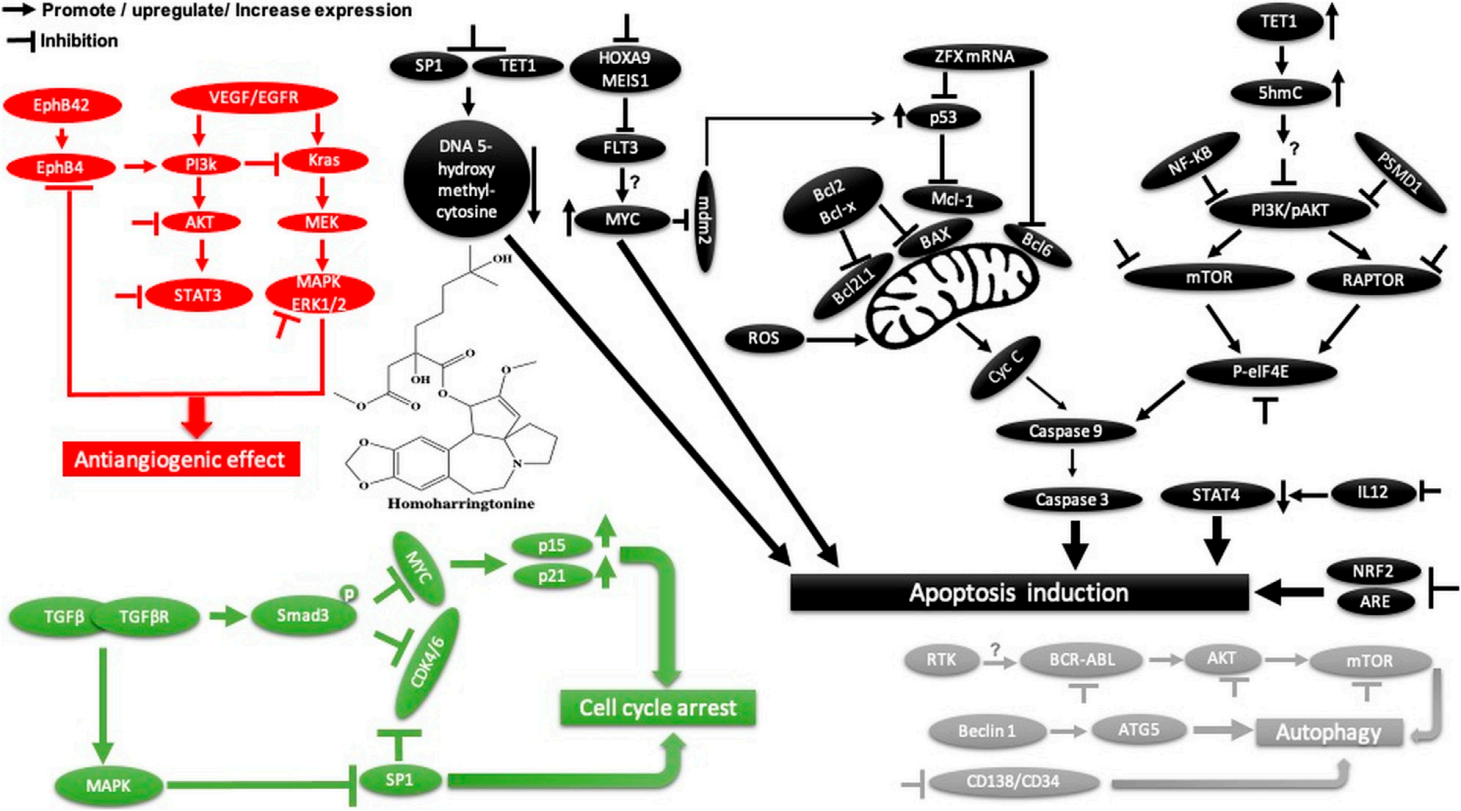
Main mechanism of homoharringtonine against cancer in inducing apoptosis, autophagy, cell cycle arrest, and angiogenesis inhibition.

Homoharringtonine, another natural plant alkaloid isolated from *Cephalotaxus* species, has been used widely for more than 30 years in traditional Chinese medicine for the treatment of hematologic malignancies, most notably acute myeloid leukemia (AML) (Table 2). It is well known that homoharringtonine potently inhibits cell growth and viability and induces cell cycle arrest and apoptosis (Figure 11), significantly inhibits disease progression *in vivo*, and substantially prolongs the survival of mice bearing murine or human AML. Interestingly, homoharringtonine treatment dramatically decreased global DNA 5-hydroxymethylcytosine abundance by targeting the SP1/TET1 axis, and TET1 depletion mimics the therapeutic effects of homoharringtonine in AML (Figure 11). Another relevant molecular mechanism explaining its potent therapeutic activity in AML involves the suppression of the SP1/TET1/5hmC/FLT3-HOXA9-MEIS1/MYC signaling pathway (C. Li et al., 2020a). Another mechanism by which homoharringtonine inhibits the activity of Sca1-positive c-kit-negative Lin-negative leukemia stem cells in murine leukemia and causes a downregulation of the c-myc signaling pathway in Kasumi-1 human leukemia cells is by directly binding to the NF-kB repressor factor. Thus, expressed genes, especially kit and c-myc, could be used as biomarkers in patients with AML under homoharringtonine treatment (X.-J. Chen et al., 2019a). Similarly, the developed polyethyleneglycol of long-circulating homoharringtonine liposomes has been shown to suppress CD138-CD34^−^ (Figure 11) multiple myeloma cancer stem cells via autophagy and apoptosis *in vitro* and *in vivo* in a xenograft mouse model (M. Li et al., 2017). Recent studies have revealed the antitumor effect of homoharringtonine against rhabdoid tumors both *in vitro* and in patient-derived xenograft tumors, which may be due to the low expression of BCL2L1 in cancer cell lines (Howard et al., 2020).

Homoharringtonine has also been reported to have cancer properties against breast cancer. It demonstrates strong cytotoxic activity against triple-negative breast cancer cell lines (MDA-MB-231) in a dose- and time-dependent manner. It has also been shown to reduce tumor growth in a mouse model at 1 mg/kg administered subcutaneously twice daily over 7 days (Yakhni et al., 2019). In chronic myelogenous leukemia (K562), homoharringtonine increases the activity of imatinib by downregulating ZFX mRNA, blocking the bcl-6/p53 pathway, and inducing apoptosis (Q. Wang et al., 2017b). In addition to the apoptotic pathway, homoharringtonine has been demonstrated to induce autophagy in imatinib-resistant K562G cells through degradation of the BCR-ABL protein mediated by p62 and silencing of the main autophagic proteins Beclin-1 and ATG5 (Figure 11) (S. Li et al., 2020b).

Another study showed that homoharringtonine significantly inhibited the NRF2- and ARE-dependent gene expression in human lung carcinoma cell lines (A549) with an IC_50_ = 12.28 nM by stabilizing the secondary structure of the guanine-rich enhancer sequence within the 5′ untranslated region (5′UTR) and sensitizes A549 tumor cells to etoposide-induced apoptosis. It is well known that etoposide sensitization triggers apoptosis by increasing the intracellular reactive oxygen species level ref 15, which may explain the cytotoxic effect of homoharringtonine toward A549 cells (Kang et al., 2019, 2).

Based on further mechanistic studies, it can be concluded that homoharringtonine induces inhibition of LoVo cell growth *in vitro* and *in vivo* through apoptosis, cell cycle arrest at the S phase, and the inhibition of the signaling pathways including MAPK-ERK1/2,PI3K-AKT and EphB4 (Shi et al., 2020). Another study has shown that homoharringtonine significantly inhibits the growth and proliferation of pancreatic cancer cells (PANC-1, MiaPaCa), induces apoptosis and activates MEK1-ERK1/2, leading to rapid expression of PSMD11, which could explain its reported cytotoxic effects (Figure 11) (L. Wang et al., 2018). Moreover, homoharringtonine induced changes in immune cell features by decreasing the expression level of IL12 and the overexpression of CD80, CD86, CD69, CD80^+^, and CD86^−^in B220 + B cells and B cells, respectively, in non-small cell lung cancer. These immunological effects were linked to the cytotoxic and anti-tumor activities of homoharringtonine via Kras-mutant gene (G12D, G12C) expression in non-small cell lung adenocarcinoma (Weng et al., 2018). It causes alternative splicing of caspase 9 and Bcl-x with increasing pro-apoptotic Bcl-xS and caspase 9b mediated by the expression of protein phosphatase 1, leading to apoptosis in various human cancer cell lines (MCF7, A549, UACC903) (Sun Q. et al., 2018).

Another mechanism of homoharringtonine in the treatment of AML is the targeting of phospho-eIF4E. Research has shown that homoharringtonine selectively reduced p-eIF4E with a decrease in McL-1 oncoprotein expression *in vitro* and *in vivo* as well (Zhou et al., 2020). In HT29, it induces apoptosis by suppressing mTOR and increasing the ratio of BAX/Bcl2, caspase 3.9, and raptor, while PI3k, AKT, and the receptor pathway were decreased. Another mechanism involved smad3 phosphorylation (Ser423/4235) to consequently affect TGF-β pathway activation, promoting cell cycle arrest at the G1 phase in AML cell lines U937 and kG-1 (J. Chen et al., 2017).

#### 4.3.2 Clinical Studies

Based on previous intense preclinical research and clinical trials, homoharringtonine has been approved by the US Food and Drug Administration (FDA) for the treatment of patients with chronic myeloid lymphoma and chronic myeloid leukemia resistance to imatinib and/or other tyrosine kinase inhibitors (Q. Wang et al., 2017b). Recently, it has been reported to be effective in patients with myelodysplastic syndrome in clinical phase II (Daver et al., 2013). Furthermore, its effect on bone marrow CD34 + CD117+ cells has been investigated in patients with chronic myelogenous leukemia (Y.-F. Li et al., 2012). Another phase II clinical study showed that homoharringtonine at 5 mg/m^2^ administered daily for 9 days by continuous infusion was found to be effective and safe for patients with relapsed or refractory acute leukemia and the blastic phase of chronic myelogenous leukemia (E. Feldman et al., 1992). However, under some circumstances, its efficacy is limited to marrow hypoplasia and prolonged pancytopenia in patients with myelodysplastic syndromes evolving to acute myeloid leukemia, compared to when it is administered in combination with hematopoietic growth factors (E. J. Feldman et al., 1996). It is noteworthy that homoharringtonine was approved by the Food and Drug Administration for the treatment of patients with chronic myeloid leukemia (Q. Wang et al., 2017b). Recently, Chen X. et al. (2019), in a randomized clinical trial, it was concluded that homoharringtonine was more efficient in the treatment of children younger than 2 years with *de novo* acute myeloid leukemia than standard regimen chemotherapy using anthracyclines. Importantly, homoharringtonine has been shown to reduce hematologic toxicity and achieve excellent event-free survival (X. Chen X.-J. et al., 2019).

#### 4.3.3 Homoharringtonine Safety

Homoharringtonine’s major toxicity is limited to the gastrointestinal tract, cardiovascular system, hematopoietic organs, and lymphoid system in rabbits, mice, and dogs ( Yongji et al., 1979; Newman, Meeks, and Denine 1981 ). The LD_50_ of homoharringtonine of CD-1 mice was 6.7 mg/kg for a single i. p. injection. However, its primary metabolite homoharringtonine acid does not appear to be toxic at doses high to 280 mg/kg. In humans, the therapeutically effective and maximum tolerated dose was 4 mg/day for acute lymphoblastic leukemia. Clinical trials regarding the toxic effects of homoharringtonine are mainly stomatitis, nausea, myelosuppression, emesis, and disturbances in cardiovascular function (Legha et al., 1984; Kantarjian et al., 2001; Ni et al., 2003). In addition, the relative efficacy and toxicity of the homoharringtonine drug may also be influenced in patients with hepatic failure. In the case of pediatric patients, homoharringtonine is well tolerated. Nevertheless, some studies have indicated that homoharringtonine use is associated with mucositis, nausea, vomiting, diarrhea, transient changes of hepatic enzymes, and hypotension (at a dose = 7 mg/m^2^ per day for up to 10 days) (Coonley et al., 1983; Neidhart et al., 1986; Luo et al., 2004).

## 5 Conclusion and Future Perspectives

Cancer is the leading cause of death and a major public health concern worldwide, and its burden is expected to rise in 2040, with 28.4 million cases (Sung et al., 2021). To avoid the serious side effects associated with traditional therapies, natural compounds, especially those isolated from plants, have been widely used in cancer prevention and/or treatment. Alkaloids isolated from plants are important chemical compounds that serve as rich reservoirs of bioactive candidate molecules for anticancer drug discovery, and many FDA-approved alkaloids currently in the market are derived from plants. In the last 5 years, some of them have shown various degrees of success in different clinical trial phases. Taken together, palmatine, sophoridine, and aleutianamine have great potential as candidate drugs that require further *in vitro* and *in vivo* investigations for treating colon, lung, and pancreatic cancers, respectively, to pave the way for clinical studies. Furthermore, both preclinical research and clinical trials establish that berberine and homoharringtonine are a promising drug against colorectal or breast and acute myeloid leukemia or breast cancers, respectively.

Several plant-based alkaloids exhibit anticancer activities at micromolar or nanomolar concentrations (Table 2), and their mechanisms of action are very complex and vary among different alkaloids. Taken together, the anticancer activity of these alkaloids against various cancer cell lines affects cell growth, cell cycle, cell invasion, angiogenesis, metastasis, autophagy, and apoptosis. Although many plant-based alkaloids demonstrate anticancer properties, the underlying molecular mechanism remains unclear, mainly the interaction between the alkaloid-binding site and the target protein.

**FIGURE 12:**
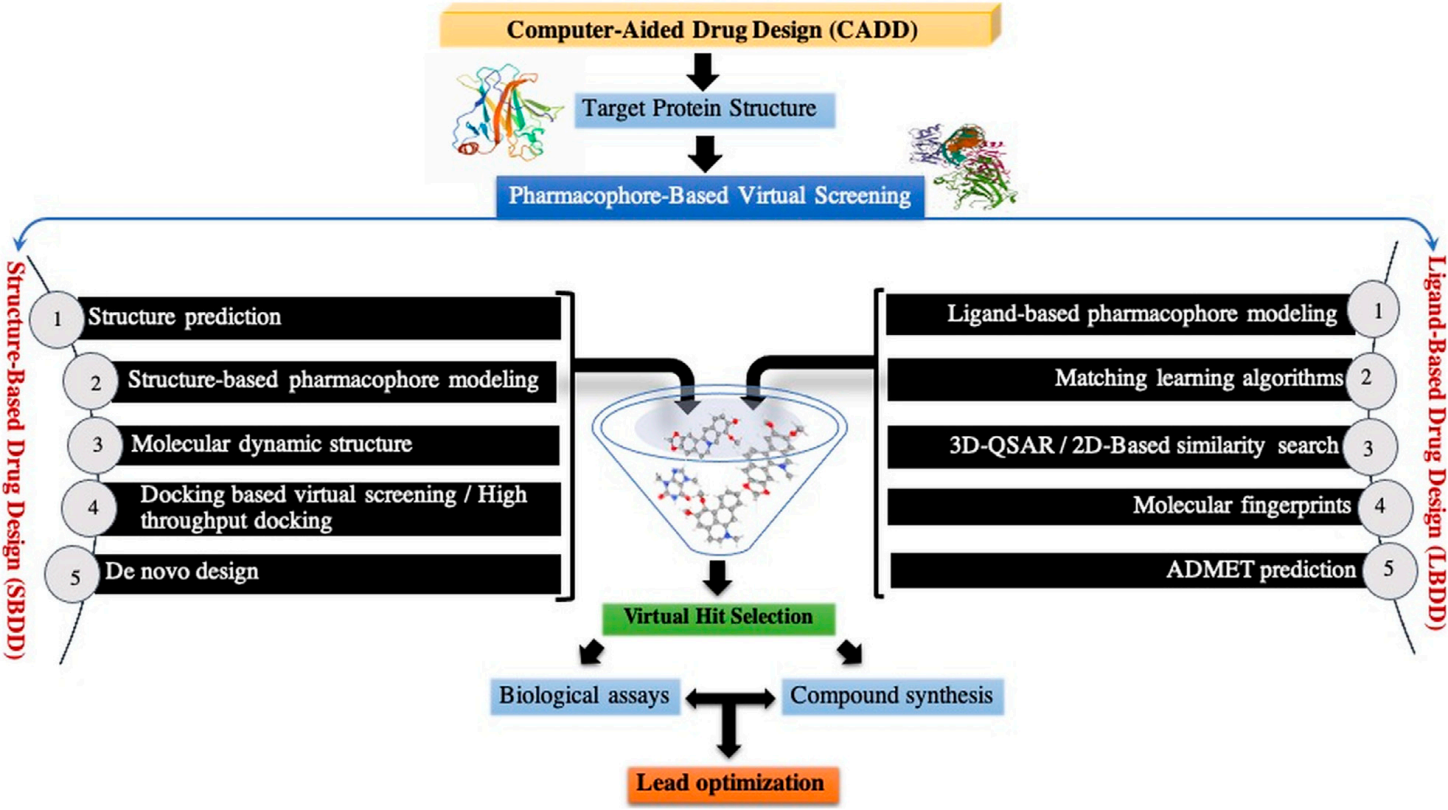
Workflow of structure- and ligand-based virtual screening approaches.

The combination of high-throughput bioinformatics-based data mining techniques and molecular assays has significantly improved our understanding of the molecular pathogenesis of cancer, classification of tumors, and now the management of cancer patients in the clinic. There are a number of bioinformatics-based approaches for mining data derived from high-throughput information-rich methods such as genomic, microarray gene (gene chip) epigenetic, genome architecture, cistromic, transcriptomic, proteomic, and ribosome profiling data have all made significant contribution for identification of molecular targets in cancer and elucidation of molecular pathways. For example, weighted gene co-expression network analysis (WGCNA) can be used with network pharmacology to predict and decipher the molecular mechanism of alkaloids in the treatment of cancer ( Yuan et al., 2020 ). Nowadays, computer-aided drug discovery and design (CADDD) computation tools are used for management, modeling, and analysis of molecules ( Song, Lim, and Tong 2009 ) at every phase of cancer-drug discovery project, from lead optimization and target validation to preclinical research ( Jorgensen 2004 ) (Figure 12).

Structure-based pharmacophore modeling and computational approaches such as quantitative structure–activity relationships (QSAR) and quantitative structure–properties relationships (QSPR) may provide useful insights into the nature of ligand-binding sites in the different targets and identifying new interaction points and novel ligands with efficient ligand–receptor binding affinity, which in consequence results in predictive models that can be adequate for drug hit discovery and applied in virtual screening, multitarget drug design, lead discovery, optimization, and in the *de novo* design ( Prada-Gracia et al., 2016 ) (Figure 11). One of the most widely applied groups of docking algorithms used in the *de novo* drug design is genetic algorithms used by docking software such as AutoDock ( Tiwari et al., 2009 ), GOLD ( Maia et al., 2020 ), GANDI ( Dey and Caflisch, 2008 ), and PhDD (pharmacophore-based *de novo* design) ( Huang et al., 2010 ).

Plant-derived alkaloids have historically been continuing and will continue to be a precious source for both cytotoxic chemotherapy and molecular-targeted cancer therapy. Sometimes, but more often, they need careful structural optimization to improve their efficacy, pharmacokinetic, and safety profiles as well as chemical accessibility. Furthermore, a deep understanding of the cross-talk between alkaloids and associated signaling pathways will be helpful to better understand their molecular mechanisms of action and pharmacokinetic performances and therefore the development of anticancer drugs that are more effective, selective, and less toxic.

## Glossary

5hmC: 5-hydroxymethylcytosine
ABCG2: ATP-binding cassette transporter G2
ABL: Abelson proto-oncogene
AIF: Apoptosis-inducing factor
AKT: Protein kinase B
AMPK: AMP-activated protein kinase
AP-2: Adapter protein complex 2
ARE: Antioxidant response element
ATG: Autophagy-related protein
AURKA: Aurora kinase A
BCR: Breakpoint cluster region gene
BCRP: Breast cancer resistance protein
COL1 A1: Collagen, type I, alpha 1
DIABLO: Direct IAP binding protein with low pI
eIF4E: Eukaryotic translation initiation factor 4E
EphB4: Ephrin type-B receptor 4
ER: Endoplasmic reticulum
ERK: Extracellular signal–regulated kinase
FAK: Focal adhesion kinase
FLICE: Fas-associated death domain-like interleukin-1β–converting enzyme
FOXO1: Forkhead box O1
GRP78: Glucose-regulated protein 78
GSK3B: Glycogen synthase kinase 3
HIF-1 ⍺: Hypoxia-inducible factor -1⍺
HMGR: 3-hydroxy-3-methylglutaryl-coenzyme A reductase
HOXA9: Homeobox A9
hTERT: Human telomerase reverse transcriptase
IAP: Inhibitor of apoptosis protein
iPLA2: Independent phospholipase A2
IRF3: Interferon-responsive factor 3
JNKs: c-Jun N-terminal kinases
LATS: Large tumor suppressor
LC3: Microtubule-associated protein 1A/1B-light chain 3
LOX-5: 5-lipoxygenase
LTB4: Leukotriene B4
MAPKs: Mitogen-activated protein kinases
MCL-1: Myeloid-cell leukemia 1
MDM2: Mouse double minute 2 homolog
Meis1: Myeloid ecotropic viral integration site 1
MMPs: Matrix metalloproteinases
mTOR: Mammalian target of rapamycin
NOSs: Nitric oxide synthases
Nrf2: Nuclear erythroid 2-related factor 2
PARPs: Poly (ADP-ribose) polymerases
PD-1: Programmed cell death 1
PDE4: Phosphodiesterase 4
PDGF: Platelet-derived growth factor
PI3Ks: Phosphoinositide 3-kinases
PSMD1: 26S proteasome non-ATPase regulatory subunit 1
PTEN: Phosphatase and tensin homolog
RANKL: Receptor activator of nuclear factor kappa-Β ligand
RAPK: Rho-associated protein kinase
RAR: Retinoic acid receptor alpha
ROCK: Rho-associated coiled-coil kinase
RPS6: Ribosomal protein S6
RTKs: Receptor tyrosine kinases
SCAP: SREBP cleavage-activating protein
SHH: Sonic hedgehog
SMAC: Second mitochondria-derived activator of caspase
SMAD3: Mothers against decapentaplegic homolog 3
Sp1: Specificity protein 1
SREBF1: Sterol regulatory element-binding protein 1
SRSF3: Ser/Arg-rich splicing factor 3
STAT3: Signal transducer and activator of transcription 3
TET1: Tet methylcytosine dioxygenase
TGF-β: Transforming growth factor beta
TLR4: Toll-like receptor 4
ULK1: Unc-51–like kinase-1
uPA: Urokinase-type plasminogen activator
VEGF: Vascular endothelial growth factor
Vps34: Vacuolar protein sorting 34
WEE1: Wee1-like protein kinase
YAP: Yes-associated protein 1
ZEB: Zinc finger e-box–binding homeobox
ZFX: Zinc finger protein x-linked
ZO1: Zonula occludens-1 (Tight junction protein-1)
